# Learning Multi-Types of Neighbor Node Attributes and Semantics by Heterogeneous Graph Transformer and Multi-View Attention for Drug-Related Side-Effect Prediction

**DOI:** 10.3390/molecules28186544

**Published:** 2023-09-09

**Authors:** Ping Xuan, Peiru Li, Hui Cui, Meng Wang, Toshiya Nakaguchi, Tiangang Zhang

**Affiliations:** 1 School of Computer Science and Technology, Heilongjiang University, Harbin 130407, China; 2 Department of Computer Science, School of Engineering, Shantou University, Shantou 515000, China; 3 Department of Computer Science and Information Technology, La Trobe University, Melbourne 3086, Australia; 4 Center for Frontier Medical Engineering, Chiba University, Chiba 263-8522, Japan; 5 School of Mathematical Science, Heilongjiang University, Harbin 130407, China

**Keywords:** drug-related side-effect prediction, multi-types of neighbor node attributes, diverse connection semantics learning, heterogeneous graph transformer, neighbor node category-level attention

## Abstract

Since side-effects of drugs are one of the primary reasons for their failure in clinical trials, predicting their side-effects can help reduce drug development costs. We proposed a method based on heterogeneous graph transformer and capsule networks for side-effect-drug-association prediction (TCSD). The method encodes and integrates attributes from multiple types of neighbor nodes, connection semantics, and multi-view pairwise information. In each drug-side-effect heterogeneous graph, a target node has two types of neighbor nodes, the drug nodes and the side-effect ones. We proposed a new heterogeneous graph transformer-based context representation learning module. The module is able to encode specific topology and the contextual relations among multiple kinds of nodes. There are similarity and association connections between the target node and its various types of neighbor nodes, and these connections imply semantic diversity. Therefore, we designed a new strategy to measure the importance of a neighboring node to the target node and incorporate different semantics of the connections between the target node and its multi-type neighbors. Furthermore, we designed attentions at the neighbor node type level and at the graph level, respectively, to obtain enhanced informative neighbor node features and multi-graph features. Finally, a pairwise multi-view feature learning module based on capsule networks was built to learn the pairwise attributes from the heterogeneous graphs. Our prediction model was evaluated using a public dataset, and the cross-validation results showed it achieved superior performance to several state-of-the-art methods. Ablation experiments undertaken demonstrated the effectiveness of heterogeneous graph transformer-based context encoding, the position enhanced pairwise attribute learning, and the neighborhood node category-level attention. Case studies on five drugs further showed TCSD’s ability in retrieving potential drug-related side-effect candidates, and TCSD inferred the candidate side-effects for 708 drugs.

## 1. Introduction

The side-effects of drugs are defined as effects occurring in the body when the drug is administered at therapeutic doses that are unrelated to its therapeutic purpose, including adverse reactions that may cause the drug to fail in clinical trials [1,2,3]. Therefore, providing precise and efficient identification of drug-related side-effect candidates can aid in lowering drug development costs and enhance drug safety [4,5]. Computational methods have demonstrated their ability to aid in drug discovery [6] and design [7] (CADD). They can also screen for reliable drug-related side-effect candidates [8,9,10].

The three categories of currently used drug-side-effect association prediction methods are as follows: The first category involves estimation of drug and side-effect association likelihoods based on drug-associated proteins. New indications and adverse reactions are usually caused by unexpected chemical–protein interactions at off-target sites. Therefore, the targeted protein information of the drug is used to predict drug-related side-effects. Compound–protein interaction (CPI) sets [11,12] and drug–protein interactions (DPI) can also be used to infer drug-related side-effect candidates [13]. However, this class of method is limited in that only a small fraction of the structural information for the drug-associated proteins is available [14].

A second class of predictive models uses machine learning to screen candidates for drug-related side-effects. To combine data on medications, proteins, and side-effects, five machine learning techniques were used: logistic regression, parsimonious Bayes, k-nearest neighbors, random forest, and support vector machine [15]. Approaches to infer potential drug-side-effect associations are based on multi-label learning [16], on multiple kernels learning and least squares [17], on random forests [18], on a random wandering and skip-gram algorithm [19], on feature-derived graph-regularized matrix factorization for predicting drug side-effects (FGRMF) [20], on triple matrix decomposition based on nuclear target alignment [21], and on non-negative matrix factorization [22]. Mohsen et al. [23] constructed a framework based on a deep neural network (DNN) for inferring the candidates. However, such models are shallow prediction models which have difficulty in fully extracting the complicated and nonlinear associations between drugs and side-effects.

The third category establishes a prediction model based on deep learning to further enhance prediction performance by extracting the depth and representative features of the drug and side-effect nodes. The training process of a deep learning model usually needs several hours or tens of hours. On the other hand, when the model is applied to inferring the association possibility for a pair of drug and side-effects, it often only needs no more than a second. The newly advanced models make full use of the diverse data related to drug and side-effect nodes for drug-side-effect association prediction, including the similarity and association information of drugs and side-effects as well as the association information of drugs and diseases. Several approaches integrate multi-source data on drugs and side-effects, including through use of graph attention networks [24], a similarity-based deep learning approach for determining the frequencies of drug side-effects (SDPred) using a multi-layer perceptron [25], graph convolutional autoencoders, and convolutional neural networks [26], respectively. Recently, hybrid graph neural network models incorporating graph-embedding and node-embedding modules have been used to model drug-side-effect associations and to provide candidate predictions [27]. Although deep models have shown improvements in drug-side-effect association predictions, the above models cannot adequately fuse the features of the edges between the source and target nodes and do not integrate the rich positional information in the feature embedding of the node pairs. Our model aggregates the information from multiple types of neighbor nodes, and encodes the semantic information of the various connections. Moreover, an attribute learning module is built to learn the pairwise attributes from a multiple capsule perspective.

In this study, we propose a novel prediction model TCSD for integrating the various neighbor attributes, the diverse connection semantics, and the pairwise attributes. TCSD’s main contributions are listed as follows:

(1) First, two heterogeneous graphs composed of drug and side-effect nodes are constructed by utilizing two types of drug similarities to complement the encoding of the specific topology structure and node attributes of each heterogeneous graph. A target node in each graph has drug neighbor nodes and side-effect nodes, and there are contextual relationship among the attributes of the target node and the attributes of its diverse neighbor nodes. Most previous approaches have focused only on aggregating the information of a single type of neighbor node. A module based on a graph transformer is established to learn category-sensitive attributes for each category of neighbor nodes.
(2) Previous approaches did not fully utilize the diverse information of multiple types of connections among the drug and side-effect nodes. In order to improve the node feature-learning capacity in each heterogeneous graph, we design a strategy to integrate the similarity semantic connections between drugs (side-effects) and the association semantic connections between drugs and side-effects.
(3) Third, we design two attention mechanisms for the effective fusion of learned information. To adaptively fuse the encoded contextual features from the drug neighbor nodes and the side-effect nodes for each target node, we design the attention at the neighbor category level. Since two heterogeneous graphs make different contributions to drug-related side-effect prediction, we design an attention from the graph perspective to discriminate their contributions.
(4) Finally, we propose a capsule network-based strategy to learn the attributes of a pair of drug and side-effect nodes. The created multiple capsules and the dynamic routing mechanism enhance position information learning in the pairwise attribute embedding. Previous approaches did not integrate the information of the positions in the pairwise embedding. A comprehensive comparison with six state-of-the-art methods and case studies on five drugs showed TCSD’s superior performance and its ability in discovering potential association candidates.

## 2. Materials and Methods

**Figure 1:**
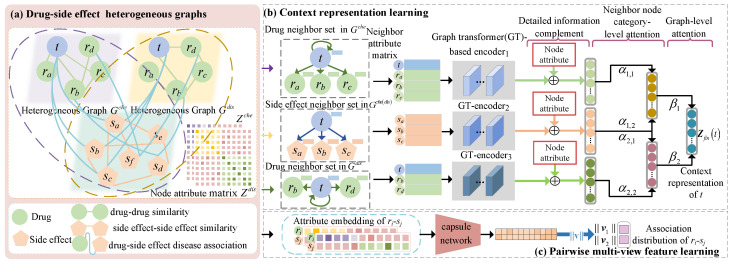
Framework of the proposed TCSD prediction model. (**a**) Establish two drug-side-effect graphs according to two types of drug similarities and demonstrate their attribute matrices (**b**) Learn the context representations of the drug and side-effect nodes based on a graph transformer and two attentions (**c**) Construct the capsule network to learn the multi-view pairwise attributes.

**Figure 2:**
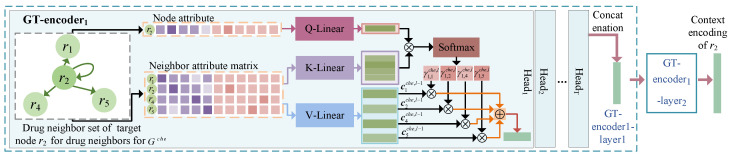
Illustration of learning the context representation based on graph transformer for a drug node.

The new prediction model TCSD is presented in Figure 1. It integrates the multi-modality similarities of medications and side-effects, neighbor context encoding, and pairwise feature representation to predict drug-related potential side-effects. First, two drug–side-effect heterogeneous graphs were created based on the associations between drugs and side-effects as well as the multi-modality similarities (Figure 1a). Afterwards, to learn the neighbor context encoding of the target node, we built a transformer-based context encoding (CET) module using a neighbor node category-level and a graph-level attention mechanism (Figure 1b) with detailed structures, as shown in Figure 2. In parallel, a capsule network-based acquisition pairwise multi-view feature (MVF) learning module (Figure 1c) was used to learn the feature map of a pair of drug–side-effect nodes.

### 2.1. Dataset

Public databases [28,29] and papers [26,30] addressing drug-side-effect associations, side-effect similarities, drug chemical substructure similarities, and drug functional similarities were used to gather data on drugs and side-effects. Initially, 80,164 pairs of drug and side-effect associations were retrieved from the SIDER databank [28]. We obtained the chemical substructural similarities from the comparative toxicogenomics database [29], which includes the chemical substructures of 708 drugs. The disease-based drug similarities were obtained from a previous study [31]. These associations and similarities included 708 drugs, 4192 side-effects, and 5603 diseases.

### 2.2. Multi-Source Data Matrix Representation and Construction of Heterogeneous Graphs

#### 2.2.1. Matrix Representation of Drug-Side-Effect Associations

We created an association matrix $A=A_{i,j}\in R^{N_{r}*N_{s}}$ according to the discovered associations of the drug-side-effect node pairs. This matrix illustrates the relationships between $N_{r}$ drugs and $N_{s}$ side-effects. The drugs are represented by the rows of *A* and the adverse effects are represented by the columns. If a drug $r_{i}$ and side-effect $s_{j}$ are known to be associated, then $A_{i,j}=1$. If not, then $A_{i,j}=0$.

#### 2.2.2. Matrix Representation of Multi-Modality Similarities of Drugs

When two drugs $r_{i}$ and $r_{j}$ are associated with a greater number of similar diseases, the functional similarity of the two drugs is usually greater. We, therefore, computed the functional similarity $D_{i,j}^{dis}$ between a pair of drug nodes $r_{i}$ and $r_{j}$ based on the diseases they are connected with, in accordance with the work of Wang et al. [31]. Similarly, a greater similarity in the chemical substructures of $r_{i}$ and $r_{j}$ indicates a greater similarity between the drugs themselves. Based on this biological premise, $D_{i,j}^{che}$ was calculated based on Luo et al. using the cosine similarity to reflect the similarity of the drug chemical substructures [30]. Using the drug-related multi-source data, we obtained a multimodal similarity matrix $D^{\rho }$ for the drug defined as

(1) $$D^{\rho }=\{\begin{array}{c} D^{dis}=D_{i,j}^{dis}\in R{}^{N_{r}*N_{r}}\\ D^{che}=D_{i,j}^{che}\in R{}^{N_{r}*N_{r}} \end{array},$$

where ρ=che or dis. $D_{i,j}^{\rho }$ is used to denote the ρth similarity of $r_{i}$ and $r_{j}$. In addition, $D_{i,j}^{\rho }\in \left[0,1\right]$. The value of $D_{i,j}^{\rho }$ increases with the degree of resemblance between $r_{i}$ and $r_{j}$.

#### 2.2.3. Matrix Representation of Side-Effect Similarity

A greater number of similar drugs being associated with side-effects $s_{i}$ and $s_{j}$ indicates a greater similarity between $s_{i}$ and $s_{j}$. We calculated the similarity matrix $S=S_{i,j}\in R{}^{N_{s}*N_{s}}$ of all side-effects based on the approach adopted by Wang et al. [26]. With a number between 0 and 1, $s_{i,j}$ indicates how similar side-effect $s_{i}$ and side-effect $s_{j}$ are to one another. The larger the similarity value, the higher the similarity between $s_{i}$ and $s_{j}$.

#### 2.2.4. Construction of Drug-Side-Effect Heterogeneous Graphs and Attribute Extraction

$D^{che}$ and $D^{dis}$ represent the similarities according to the chemical substructures of the two drugs and diseases that they are associated with, respectively. We created two drug-side-effect heterogeneous graphs relying on $D^{che}$ and $D^{dis}$, respectively, where ρ=che or dis. The set of nodes $V=\left\{V^{r}\cup V^{s}\right\}$ in each heterogeneous graph comprises the set of drug nodes $V^{r}$ and the set of side-effect nodes $V^{s}$; an edge $e_{i,j}^{\rho }\in E^{\rho }$ with a weight $w_{i,j}^{\rho }\in W^{\rho }$ links a pair of nodes $v_{i}$, $v_{j}$. In general, several types of connecting edges can exist between drugs and side-effects, including a drug–side-effect association edge $e_{rs}$, a drug–drug similarity edge $e_{rr}$, and a side-effect–side-effect similarity edge $e_{ss}$. $W^{\rho }$ contains the association matrix *A* and similarity matrices *S* and $D^{\rho }$. The adjacency matrix of the ρth heterogeneous graph is expressed as $I^{\rho }$,

(2) $$I^{\rho }=\left[\begin{array}{cc} D^{\rho } & A\\ A^{T} & S \end{array}\right]\in R{}^{N_{total}*N_{total}},$$

where the total number of nodes is $N_{total}=N_{r}+N_{s}$ and $A^{T}$ denotes the transpose of the matrix *A*. The *i*-th row in the matrix $I^{\rho }$ denotes the association and similarity of the node $v_{i}$ with all of the drugs and side-effects, which are considered as node attributes of $v_{i}$. The attribute vector $x_{i}^{\rho }$ of the drug $r_{i}$ is defined as

(3) $$x_{i}^{\rho }=\left[D_{i,}^{\rho }\|A_{i,}\right]\in R{}^{N_{total}},$$

where ρ=che or dis, and ‖ indicates the operation of the first and last link. The *i*-th row of the matrix *A*, where each side-effect’s association with $r_{i}$ is recorded, is designated by the symbol $A_{i,}$. $D_{i,}^{che}\left(D_{i,}^{dis}\right)$ is the row *i* of the matrix $D^{che}\left(D^{dis}\right)$ containing the chemical substructural (functional) similarities with all drugs.

Similarly, the attribute vector of the side-effect $s_{j}$ is represented as $y_{j}$,

(4) $$y_{j}=\left[A_{,j}\|S_{,j}\right]\in R{}^{N_{total}},$$

where $A_{,j}\left(S_{,j}\right)$ denotes the connection with the association (similarity) of $s_{j}$ and all drugs (side-effects). The feature embedding matrix $Z^{\rho }$ of the node pairs $r_{i}$ and $s_{j}$ is defined as

(5) $$Z^{\rho }=\left[\begin{array}{c} x_{i}^{\rho }\\ y_{j} \end{array}\right]=\left[\begin{array}{cc} D_{i,\alpha }^{\rho } & A_{i,\alpha }\\ A_{\alpha ,j} & S_{\alpha ,j} \end{array}\right]\in R{}^{2*N_{total}},$$

where $2*N_{total}$ is the dimension of $Z^{\rho }$.

### 2.3. Context Representation Learning Based on Transformer with Attention

The target node attributes are contextually linked to the attributes of the neighbors of each category in their neighborhood. In order to learn the context representations of the nodes, we designed the CET module based on a graph-level attention mechanism to aggregate information regarding its neighbor nodes. As each heterogeneous graph has a unique topology, we used a graph transformer (GT) module (Figure 2) for $G^{che}$ and $G^{dis}$. The semantic information of the similarity or association connection edges between the neighbor node and target node was used to learn the corresponding neighborhood context representation. The module comprised $l_{e}$ coding levels; layer *l* can serve as an illustration of how the context is learned. The CET module’s drug node and side-effect node learning processes were similar; an example is described for drug $r_{i}$.

#### 2.3.1. Neighborhood Node Set Extraction

Based on the similarity between the drug $r_{i}$ and all drugs, we obtained the top $N_{t}$ most similar neighbors to $r_{i}$. If $N_{t}=4$, let $r_{i}$, $r_{a}$, $r_{b}$, and $r_{c}$ be the four top neighbor nodes, and their attribute vectors be $x_{i}^{\rho }$, $x_{a}^{\rho }$, $x_{b}^{\rho }$, and $x_{c}^{\rho }$, respectively. The set of attribute vectors of the drug neighbor nodes of $r_{i}$ is denoted as $S_{r_{i},r}$,

(6) $$S_{r_{i},r}=\left\{x_{i}^{\rho },x_{a}^{\rho },x_{b}^{\rho },x_{c}^{\rho }\right\}.$$

Similarly, we can obtain all of the $N_{k}$ side-effect neighbor nodes associated with $r_{i}$. When $N_{k}=3$, the $N_{k}$ side-effect neighbors of $r_{i}$ are $s_{a}$, $s_{b}$, and $s_{c}$, with $y_{a}$, $y_{b}$, and $y_{c}$ being their attribute vectors, respectively. Thus, the set of attribute vectors of the side-effect neighbor nodes of $r_{i}$ is represented as $S_{r_{i},s}$,

(7) $$S_{r_{i},s}=\left\{y_{a},y_{b},y_{c}\right\}.$$

#### 2.3.2. Node Attribute Conversion

$S_{r_{i},r}=\left\{x_{i}^{\rho },x_{m}^{\rho },m=a,b,c\right\}$ is the set of drug-like neighbor node attribute vectors for $r_{i}$. Inspired by Transformer, we mapped the attribute vector $x_{i}^{\rho }$ of $r_{i}$ to a query vector space and $S_{r_{i},r}$ to a key vector space and value vector space. To reduce the bias in the contextual semantic learning process, we established a multi-headed attention mechanism. In the *t*-th attention head, because each drug-like neighbor contributes differently to $r_{i}$, we employed a neighbor node-level attention mechanism to calculate the attention weights of $r_{i}$ for each neighbor. The output query vectors of the layer 1 and layer *l* coding layers are $q_{t}^{\rho ,1}\left(r_{i}\right)\in R{}^{n}$ and $q_{t}^{{}^{\rho ,l}}\left(r_{i}\right)\in R{}^{n}$, respectively. $q_{t}^{\rho ,1}\left(r_{i}\right)\in R{}^{n}$ and $q_{t}^{{}^{\rho ,l}}\left(r_{i}\right)\in R{}^{n}$ are calculated as follows,

(8) $$q_{t}^{\rho ,1}\left(r_{i}\right)=W_{t,Q}^{1}\cdot x_{i}^{\rho }$$

(9) $$q_{t}^{\rho ,l}\left(r_{i}\right)=W_{t,Q}^{l}\cdot c^{\rho ,l-1}\left(r_{i}\right),l=2,\ldots ,l_{e}$$

where $W_{t,Q}^{1}\in R{}^{n*N_{total}}$ and $W_{t,Q}^{l}\in R{}^{n*N_{total}}$ are the weight matrices of layer 1 and layer *l*, respectively. $c_{i}^{\rho ,l-1}$ is the vector of the encoded information of $r_{i}$ obtained in layer l−1; $l_{e}$ is the number of layers of the encoding layer. We calculate the key matrix $K_{t}^{\rho ,l}\in R{}^{4*n}$ and value matrix $V_{t}^{\rho ,l}\in R{}^{4*n}$ for $r_{i}$ as follows:

(10) $$K_{t}^{\rho ,l}=W_{t,K}^{l}{\left[c_{i}^{\rho ,l-1}\|c_{m}^{\rho ,l-1}\right]}^{T},l=1,2,\ldots ,l_{e}$$

(11) $$V_{t}^{\rho ,l}=W_{t,V}^{l}{\left[c_{i}^{\rho ,l-1}\|c_{m}^{\rho ,l-1}\right]}^{T},l=1,2,\ldots ,l_{e}$$

where $W_{t,K}^{l}$ and $W_{t,V}^{l}$ are the weight matrices. ‖ represents the splicing between two vectors. $c_{i}^{\rho ,l-1}$ and $c_{m}^{\rho ,l-1}$ are the results of the layer l−1 encoding of $r_{i}$ and its neighbors, respectively, and $c_{i}^{\rho ,0}$ and $c_{m}^{\rho ,0}$ are their attribute vectors $x_{i}^{\rho }$ and $x_{m}^{\rho }$, respectively.

#### 2.3.3. Contextual Encoding of Nodes of the Same Type

All of the drug-type neighbor nodes of drug $r_{i}$ form the set $\left\{r_{i},r_{m},m=a,b,c\right\}$, and a contextual connection exist between the node properties of $r_{i}$ and the properties of these neighbor nodes. Therefore, we must gather information about the neighbors of $r_{i}$ to update the attribute vector of $r_{i}$. We calculate the attention score of $r_{v}$ to $r_{i}$ as $\alpha_{t}^{\rho ,l}\left(r_{i},r_{v}\right)$,

(12) $$\alpha_{t}^{\rho ,l}\left(r_{i},r_{v}\right)=K_{t}^{\rho ,l}W_{t,D}^{l}\cdot q_{t}^{\rho ,l}{\left(r_{i}\right)}^{T},$$

where v=i,a,b or *c*. $W_{t,D}^{l}\in R{}^{n*n}$ is a weight matrix specific to the drug-like neighbor nodes of $r_{i}$ for fusing the corresponding semantic information for each connection (similarity connection or association connection). Then, for the neighborhood nodes $r_{i}$, $r_{a}$, $r_{b}$, and $r_{c}$ of $r_{i}$, and the obtained $\alpha_{t}^{\rho ,l}\left(r_{i},r_{i}\right)$, $\alpha_{t}^{\rho ,l}\left(r_{i},r_{a}\right)$, $\alpha_{t}^{\rho ,l}\left(r_{i},r_{b}\right)$, and $\alpha_{t}^{\rho ,l}\left(r_{i},r_{c}\right)$, the normalized attention weight is obtained as $\gamma_{t,v}^{\rho ,l}$,

(13) $$\gamma_{t,v}^{\rho ,l}=\frac{\exp(\alpha_{t}^{\rho ,l}\left(r_{i},r_{v}\right))}{\sum_{j\in \left\{i,a,b,c\right\}}\exp(\alpha_{t}^{\rho ,l}\left(r_{i},r_{j}\right))},$$

where exp is an exponential function. The drug-like neighbor encoding information $y_{t,e_{rr}}^{\rho ,l}\left(r_{i}\right)$ of $r_{i}$ can be represented as,

(14) $$y_{t,e_{rr}}^{\rho ,l}\left(r_{i}\right)=\sum_{v\in \left\{i,a,b,c\right\}}\gamma_{t,v}^{\rho ,l}V_{t}^{\rho ,l}\left(r_{v}\right),$$

where $y_{t,e_{rr}}^{\rho ,l}\left(r_{i}\right)\in R{}^{n}$. Finally, the context encoding $y_{e_{rr}}^{l}\left[r_{i}\right]\in R{}^{nT}$ at the drug neighbor node level of $r_{i}$ is defined as,

(15) $$c^{\rho ,l-1}\left(r_{i}\right)=y_{e_{rr}}^{\rho ,l}\left(r_{i}\right)=\overset{T}{\underset{t=1}{\|}}y_{t,e_{rr}}^{\rho ,l}\left(r_{i}\right),$$

where ‖ denotes the first and last join of the T-head attention encoding vector. Similarly, for the set $\left\{s_{a},s_{b},s_{c}\right\}$ of the side-effect neighbor nodes of $r_{i}$, we can obtain the context encoding $y_{e_{rs}}^{\rho ,l}\left(r_{i}\right)$ specific to that class of neighbor nodes.

#### 2.3.4. Neighborhood Node Category-Level and Graph-Level Attention Mechanisms

Since the drug node $r_{i}$ has two types of neighbor nodes, which are drug and side-effects, we learn the context encodings $y_{e_{rr}}^{\rho ,l}\left(r_{i}\right)$ and $y_{e_{rs}}^{\rho ,l}\left(r_{i}\right)$ of $r_{i}$, respectively. As $y_{e_{rr}}^{\rho ,l}\left(r_{i}\right)$ and $y_{e_{rs}}^{\rho ,l}\left(r_{i}\right)$ differ in their learning contributions to the final contextual representations of $r_{i}$, we propose a neighborhood node category-level attention mechanism. The attention score is obtained as,

(16) $$s_{u,nei}^{\rho ,l}=h_{nei}^{\rho ,l}\tanh\left(W_{u,nei}^{\rho ,l}y_{e_{ru}}^{\rho ,l}\left(r_{i}\right)+b_{nei}^{\rho ,l}\right),$$

where $u\in \left\{r,s\right\}$, $W_{u,nei}$ is the weight matrix of the first-class neighbor nodes; $h_{nei}^{\rho ,l}$ and $b_{nei}^{\rho ,l}$ are the weight and bias vectors, respectively. The normalized attention score is calculated as $\beta_{r_{i},u}^{\rho ,l}$,

(17) $$\beta_{r_{i},u}^{\rho ,l}=\frac{\exp(s_{u,nei}^{\rho ,l})}{\sum_{j\in \left\{r,s\right\}}\exp(s_{j,nei}^{\rho ,l})}.$$

The contextual encoding of $r_{i}$, as enhanced by the attention mechanism, is obtained as $Z_{con}^{\rho ,l}\left(r_{i}\right)$,

(18) $$Z_{con}^{\rho ,l}\left(r_{i}\right)=\sum_{u\in \left\{r,s\right\}}\beta_{r_{i},u}^{\rho ,l}y_{e_{rr}}^{\rho ,l}\left(r_{i}\right),$$

where $Z_{con}^{\rho ,l}\left(r_{i}\right)\in R{}^{nT}$. The encoding result $Z_{con}^{\rho ,l_{e}}\left(r_{i}\right)\in R{}^{n_{fin}}$ obtained by the $l_{e}$-th layer GT contains contextual information regarding the two types of neighbor nodes of $r_{i}$ in the heterogeneous graph $G^{\rho }$ with the discriminative semantics of the connected edge; it is renamed as $Z^{\rho }\left(r_{i}\right)$.

$x_{i}^{\rho }$ contains more detailed information and $Z^{\rho }\left(r_{i}\right)$ carries out learning to obtain the representative neighborhood contextual encoding. Therefore, we added the information from $x_{i}^{\rho }$ to $Z^{\rho }\left(r_{i}\right)$. Given the original attribute vector $x_{i}^{\rho }$ of $r_{i}$, we first applied a linear projection $S-Linear^{\rho }$ to map it to the attribute space of $Z^{\rho }\left(r_{i}\right)$. Then, we superimposed it with $Z^{\rho }\left(r_{i}\right)$ to obtain a complemented neighbor context encoding as $Z_{add}\left(r_{i}\right)$,

(19) $$Z_{add}^{\rho }\left(r_{i}\right)=S-Linear^{\rho }\left(\sigma \left(x_{i}^{\rho }\right)\right)+Z^{\rho }\left(r_{i}\right),$$

where σ is the relu activation function [32].

The heterogeneous graphs $G^{che}$ and $G^{dis}$ were learned by the CET module to obtain the contextual encodings of $r_{i}$ and $s_{j}$ represented as $Z_{add}^{\rho }\left(r_{i}\right)$ and $Z_{add}^{\rho }\left(s_{j}\right)$ (ρ=che or dis), respectively. $Z_{add}^{che}\left(r_{i}\right)\left(Z_{add}^{dis}\left(r_{i}\right)\right)$ and $Z_{add}^{che}\left(s_{j}\right)\left(Z_{add}^{dis}\left(s_{j}\right)\right)$ were stacked up and down to form $Z_{add}^{che}\left(r_{i}-s_{j}\right)\in R{}^{2*n_{fin}}\left(Z_{add}^{dis}\left(r_{i}-s_{j}\right)\right)$. $Z_{add}^{che}\left(r_{i}-s_{j}\right)$ and $Z_{add}^{dis}\left(r_{i}-s_{j}\right)$ were fused by 1 × 1 convolution to form a contextual representation $Z_{fin}\left(r_{i}-s_{j}\right)\in R{}^{2*n_{fin}}$ of the node pair. $Z_{fin}\left(r_{i}\right)$ and $Z_{fin}\left(s_{j}\right)$ were spliced first and last, respectively, to form a feature map $Z_{i,j}\in R{}^{2n_{fin}}$ of $r_{i}-s_{j}$ node pair. $y_{CET}$ denotes the probability distribution of whether $r_{i}$ and $s_{j}$ are related,

(20) $$y_{CET}=softmax\left(W_{f}Z_{i,j}+b_{f}\right),$$

where $W_{f}$ is the weight matrix and $b_{f}$ is the bias vector. $y_{CET}=\left(y_{{}_{CET}}^{0},y_{{}_{CET}}^{1}\right)$, where $y_{{}_{CET}}^{0}$ is the probability that the drug $r_{i}$ and side-effect $s_{j}$ are not associated and $y_{{}_{CET}}^{1}$ is the probability that they are associated.

### 2.4. Local Information Enrichment Strategy for Drug-Side-Effect Node Pair Feature Representation Learning Based on Capsule Networks

**Figure 3:**
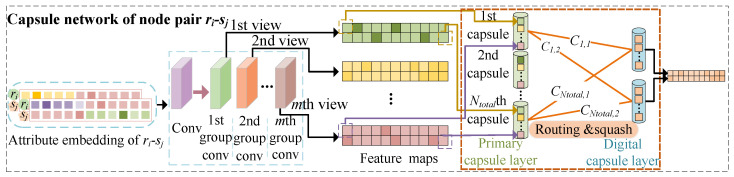
Explanation of learning pairwise multi-view features of drug-side-effect node pair with capsule networks.

Given $Z^{\rho }\in R{}^{2*N_{total}}$, which contains information regarding the similarity and association of $r_{i}$ and $s_{j}$ with all drugs and side-effects and contains $2*N_{total}$ elements, we built the MVF capsule network-based module to deeply integrate the characteristics of multiple elements at the same position from multiple views. These characteristics formed a capsule, and all newly created capsules passed through a routing mechanism to further evaluate the association scores of node pairs. The MVF module contained two convolutional layers and two capsule layers. The detailed architecture is given in Figure 3.

#### 2.4.1. Establishment of Primary Capsule Embedding Based on Convolution Operation

The feature-embedding matrices of a node pair $r_{i}$ and $s_{j}$ in the heterogeneous graphs $G^{che}$ and $G^{dis}$ are $Z^{che}$ and $Z^{dis}$, respectively. $Z^{che}$ and $Z^{dis}$ were stacked up and down to form the node pair feature-embedding matrix $Z\in R{}^{2*2*N_{total}}$ of $r_{i}$ and $s_{j}$. *Z* was fed to the convolution module to form the embedding of the primary capsule network. The convolution module contained one layer of single-group convolutional layers and one layer of multi-group convolutional layers. In the first convolutional layer, we applied a one-round zero-fill operation on *Z* to create a new matrix $\overset{\wedge }{Z}$ for learning the edge information. $l_{f}$ and $w_{f}$ were the length and width of the filter, respectively. If the number of filters was $n_{f}$, the filter $W_{conv1}\in R{}^{l_{f}*w_{f}*n_{f}}$ was applied to the matrix $\overset{\wedge }{Z}$ and the feature map $Z_{conv1}\in R{}^{n_{f}*\left(4-w_{f}+1\right)*\left(2+N_{total}-l_{f}+1\right)}$ is obtained as,

(21) $$\begin{array}{c} Z_{conv1,k}\left(i,j\right)=f\left(W_{conv1}\left(k,:,:\right)*{\overset{\wedge }{Z}}_{k,i,j}+b_{conv1}\left(k\right)\right),\\ i\in \left[1,4-w_{f}+1\right],j\in \left[1,2+N_{total}-l_{f}+1\right],k\in \left[1,n_{f}\right] \end{array}$$

where *f* is the relu activation function [32] and $b_{conv1}$ is the bias vector. $Z_{conv1,k}\left(i,j\right)$ is the element of the *i*-th row and *j*-th column of the *k*-th feature map $Z_{conv1,k}$. $\overset{\wedge }{Z}\left(i,j\right)$ is the element of the matrix $\overset{\wedge }{Z}$ in row *i* column *j*. When the *k*-th filter slides to position $\overset{\wedge }{Z}\left(i,j\right)$, the region inside the filter is ${\overset{\wedge }{Z}}_{k,i,j}$, which can be calculated as,

(22) $${\overset{\wedge }{Z}}_{k,i,j}=\overset{\wedge }{Z}\left(i:i+w_{f},j:j+l_{f}\right),{\overset{\wedge }{Z}}_{k,i,j}\in R{}^{w_{f}*l_{f}}.$$

We build the *w*-group convolution in the second layer. Each group of convolution can be considered as a view of the feature map, and the attributes of the node pairs can be learned from multiple views. The filter size in each set of convolutions was $W_{conv2}\in R{}^{2*2}$, and $Z_{conv1}$ was fed to the second convolutional layer to form $Z_{conv2}^{w}\in R{}^{w*2*N_{total}}$.

#### 2.4.2. Creation of the Primary Capsule Layer

We encapsulated the value $Z_{conv2}^{1}\left(p\right),Z_{conv2}^{2}\left(p\right),\ldots ,Z_{conv2}^{w}\left(p\right)$ of the *p*-th $\left(p=1,2,\ldots ,2*N_{total}\right)$ position on the *w* feature maps $Z_{conv2}^{1}$, $Z_{conv2}^{2}$, …, $Z_{conv2}^{w}$ into a capsule to form $u_{p}\in R{}^{w}$. This capsule contained information regarding multiple views in the local area when the filter was slid into the *p*-th position of the feature map $Z_{conv1}$.The primary capsule layer contained $\left[2*N_{total}\right]$ capsules of *w*-dimensional vectors.

#### 2.4.3. Design of Capsule Layer Routing Mechanism

We used primary and digital capsule layers to build the MVF module. The digital capsule layer consisted of $n_{qn}$$n_{qd}$-dimensional prediction capsules $v_{q}\left(q=1,2,\ldots ,n_{qn}\right)$; all of these capsules received input from all of the primary capsules $u_{p}\left(p=1,2,\ldots ,2*N_{total}\right)$ of the previous layer. We implemented the delivery of location information from the primary capsule layer to the digital capsule layer by means of weights determined by the routing mechanism. First, $u_{p}$ was used to determine the correlation between the two layers by multiplying by the weight matrix $W_{pq}$ to obtain the vector as ${\hat{u}}_{q|p}\in R{}^{n_{pd}}$,

(23) $${\hat{u}}_{q|p}=W_{pq}u_{p}.$$

${\hat{u}}_{q|p}$ was fed into the prediction capsule $v_{q}$ based on the coupling coefficients $c_{pq}$ as determined by the dynamic routing process, which were proportional to the weights of the features. We performed a dynamic routing process $n_{dr}$ times to compute $c_{pq}$. We first initialized the weight $b_{pq}=0$ between capsule *p* and capsule *q*. Next, the coupling coefficient $c_{pq}$ was obtained by normalizing the weights $b_{pq}$ with Softmax and the output vector $o_{q}$ was generated by weighted summation; $c_{pq}$ and $o_{q}$ are represented as,

(24) $$c_{pq}=\frac{\exp(b_{pq})}{\sum_{k\in \left\{1,2,\ldots ,n_{pn}\right\}}\exp(b_{pk})}$$

(25) $$o_{q}=\sum_{p}c_{pq}{\hat{u}}_{q|p}$$

The modulus lengths of $o_{q1}$ and $o_{qn_{pn}}$ were used as the uncorrelated and correlated fractions between $r_{i}$ and $s_{j}$, respectively. $o_{q}$ was employed after a nonlinear compression function to produce an output capsule $v_{q}$ as,

(26) $$v_{q}=\frac{\|o_{q}{\|}^{2}}{1+\|o_{q}{\|}^{2}}\cdot \frac{o_{q}}{\|o_{q}\|},$$

where the value of the modulus length $v_{q}$ is between 0 and 1. The update rules for $b_{pq}$ are as follows:

(27) $$b_{pq}\leftarrow b_{pq}+{\hat{u}}_{q|p}⊙v_{q},$$

where ⊙ denotes the dot product operation of two vectors. The routing mechanism is completed once after updating $b_{pq}$. After $n_{dr}$ updates, the coupling coefficients $c_{pq}$ are finally determined and the final prediction capsules $v_{q}^{fin}$ are formed. The modulus length of each vector is passed through the Softmax layer to obtain the associated probability distribution $y_{NMF}^{q}$ as,

(28) $$y_{MVF}^{q}=\frac{\exp(\|v_{q}\|)}{\sum_{k\in \left\{1,2,\ldots ,n_{pn}\right\}}\exp(\|v_{k}\|)}.$$

The prediction scores were evaluated based on the modulus length and the scores $y_{MVF}=\left[y_{MVF}^{1},y_{MVF}^{n_{pn}}\right]$ were associated with probability distributions, including the probabilities that the drug-side-effect node pair was not associated and that they were associated.

### 2.5. Final Integration and Optimization

The cross-entropy between the true label *z* and predicted association probability $y_{CET}$ was defined as the loss function when the prediction is based on the node neighbor context encoding, as follows,

(29) $$LOSS{}_{CET}=-\sum_{i=1}^{N_{train}}\sum_{j=1}^{c}z_{i}\log\left(y_{CET},j\right),$$

where $N_{train}$ is the number of training sample sets. The predicted results are classified as relevant and irrelevant $\left(c=2\right)$. The true label $z_{i}=1\left(z_{i}=0\right)$ represents the true correlation (uncorrelated) between all drugs and side-effects. In the MVF module, the cross-entropy-based loss $LOSS{}_{MVF}$ is defined as,

(30) $$LOSS{}_{MVF}=-\sum_{i=1}^{N_{train}}\sum_{j=1}^{c}z_{i}\log\left(y_{MVF},j\right).$$

We used the Adam algorithm [33] to optimize the loss functions $LOSS{}_{CET}$ and $LOSS{}_{MVF}$. Finally, a weighted sum of $y_{CET}$ and $y_{MVF}$ was calculated to obtain the final predicted association score as *y*,

(31) $$y=\gamma \times y_{CET}+\left(1-\gamma \right)y_{MVF},$$

where $\gamma \in \left(0,1\right)$ is a hyperparameter for adjusting the two knowledge contributions.

## 3. Experimental Evaluations and Discussion

### 3.1. Parameter Settings and Evaluation Metrics

TCSD was implemented in the Pytorch framework using a graphics processing unit (Nvidia GeForce GTX 2080Ti). For the CET module, the number of neighbor nodes per class $N_{t}=N_{k}=10$, the number of coding layers $l_{e}=2$, and the number of heads for the multi-headed attention was set as 8. The two encoding layers’ output feature dimensionalities were 2400 and 2000. In the MVF module, the first convolutional layer included 64 filters, while the second layer had w=8 groups of convolutions, the number of filters was 512, and the size of all the filter kernels was set to 2×2. The numbers of capsules in the initial and digital capsule layers were 4900 and 2, respectively. The dimensionality of each digital capsule was set to 32 and the number of routing mechanism iterations $n_{dr}=3$. The parameter γ at final fusion was set to 0.3.

Each prediction model’s effectiveness was evaluated using five-fold cross-validation. The positive case samples were those where the drug-side-effect associations were known and the negative case samples were the unobserved associations. As a result, we obtained 80,164 known associations betweeen drug and side-effect and 2,887,772 unknown associations. All positive case samples were divided at random into five equal parts: four of each multiple were used to train the prediction model, whereas the rest of the positive case sample set was used for testing. Randomly chosen counterexamples were used for testing, with the remaining counterexamples being used for training an array of counterexamples equal to the amount of samples in the training set that were positive.

The evaluation metrics include the area under the receiver operating characteristic (ROC) curve (AUC) [33,34], the area under the precision-recall (PR) curve (AUPR) [35], and the maximum *k* recall. The ratio of known associations to unobserved associations was approximately 1:36; evidently, a significant category imbalance existed between them. Thus, the AUPR was also used to evaluate the predictive performance as being more informative than the AUC. We determined the top $k\in \left[30,60,\ldots ,240\right]$ candidates’ recall rates as another measure of the model performance because biologists typically select drug-side-effect pairs from among these candidates and perform further relevant experiments.

### 3.2. Ablation Experiment

We conducted a series of ablation experiments to evaluate the contribution of the CET module, MVF module, and neighborhood node category-level attention mechanism (NCA) (Table 1). First, we removed the attention mechanism that was utilized to fuse the neighbor context encodings of multiple types of neighbor nodes for the target node. We performed vector summation to obtain the context representation of the target node. Next, we trained each of the two modules (CET and MVF) to obtain the contextual representation and the pairwise attributes. The attribute vectors of a pair of drug and side-effect nodes were concatenated and then went through a fully connected network to obtain the association score. The complete model with the CET module, MVF module, and NCA obtained the highest AUC=0.977 and AUPR=0.351. In the absence of the CET module, the prediction performance decreased by 1.4% in the AUC and 14.2% in the AUPR compared to TCSD. In the absence of the rich local features obtained by the MVF module, the AUC decreased by 0.6% and the AUPR decreased by 9.7% relative to TCSD. Without the NCA, the contribution of the contextual encoding to improving the prediction performance was the largest; the main reason for this was that the Transformer-based encoding strategy can propagate the node properties between the drug and side-effect nodes, thereby learning the contextual information between nodes. The MVF module learns the second most important contribution of the node pair feature representation to the results and enriches the local information of the node pairs in the process of building capsules. Accordingly, the routing mechanism can better learn the importance of the capsules.

### 3.3. Comparison with Other Methods

The six most advanced approaches were compared to our model (TCSD) in order to anticipate the drug-side-effect associations: GCRS [26], idse-HE [27], SDPred [25], Galeaon’s method [21], random walk-signed heterogeneous information network (RW-SHIN) [19], Ding’s method [17] and feature-derived graph regularized matrix factorization (FGRMF) [20]. For a fair comparison, the hyperparameters of each model were set with the same parameters as suggested in each study. The training and testing time of TCSD and the compared methods are listed in the Supplementary Table S2.

**Figure 4:**
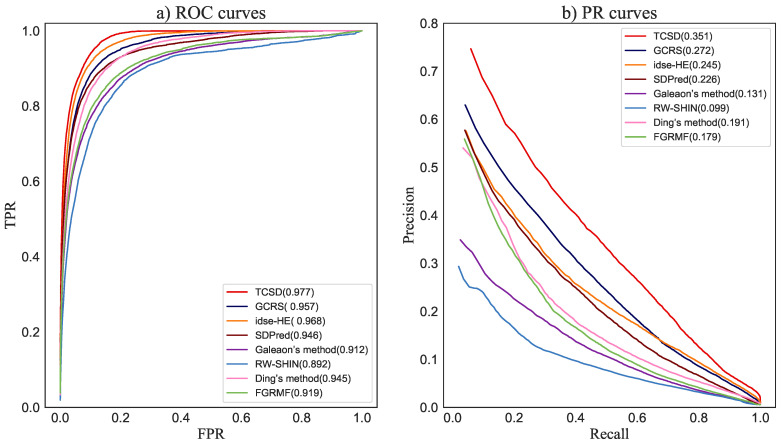
ROC curves and PR curves of our method and the compared methods for drug-side-effect association prediction.

For each drug, we calculated the corresponding AUC and AUPR in each multiple and then took the average value for the five-fold crossover as the final prediction result. The average values of the AUC and AUPR for 708 drugs were taken as the prediction performance of the entire method. As shown in Figure 4, TCSD obtained the highest AUC of 0.977, i.e., 0.9% and 2.0%, respectively, higher than idse-HE and GCRS, 3.1% and 3.2% better than SDPred and Ding’s method, respectively, 5.8% higher than FGRMF, 6.5% better than Galeaon’s method, and 8.5% higher than RW-SHIN, the worst-performing method. For the mean AUPR of all drugs, TCSD obtained the best mean AUPR value of 0.351, i.e., 7.9%, 12.5%, 16.0%, 17.2%, 22.0%, and 25.2% higher than the values from the above methods, respectively.

Idse-HE did not perform as well as our method—the possible reason is that it ignored the semantic information of the various connections in the heterogeneous graph. Our approach and GCRS both achieved good performance, primarily because we built multiple heterogeneous graphs and built an independent learning module for each heterogeneous graph. This suggests that separately learning the topological information specific to each heterogeneous graph is necessary for improving the prediction accuracy. SDPred, which is based on a multi-layer perceptron, and Ding’s method, which is based on central kernel-aligned multicore learning, both scored lower than GCRS. One possible reason for this is that both methods do not consider the topological structure in the drug-side-effect heterogeneous graphs. In addition, FGRMF and Galeaon’s method had similar AUC and AUPR values, with somewhat worse performance than the fourth-best, Ding’s method. One possible reason is that both are shallow prediction models constructed using matrix decomposition-based methods; these cannot dig deeper into the complex connections between drugs and side-effects. The performance of RW-SHIN was inferior to the other methods because it only builds a network of drug nodes without considering the topological information between side-effect nodes.

For the 708 AUCs (AUPRs) results for all prediction methods for the 708 drugs, we used 708 paired results for comparing TCSD with another method as calculated using pairs of Wilcoxon tests. With a *p*-value threshold of 0.05, the data demonstrated that TCSD significantly outperformed the other six approaches (Table 2).

**Figure 5:**
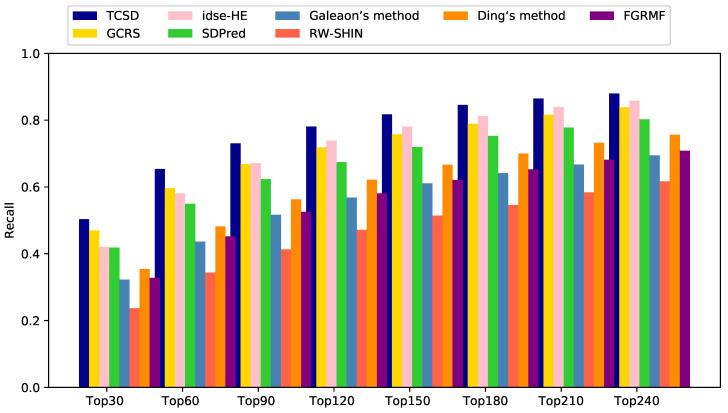
Recall rates of all the prediction methods at various top *k* values.

For the top *k* drug candidates with side-effects, a higher recall indicates that more real drug and side-effect associations are included in these candidates. Our TCSD model consistently outperformed other methods at different *k* thresholds and ranked 50.3% of the positive cases in the top 30 candidates, 65.4% in the top 60, 73.0% in the top 90, and 78.1% in the top 120. GCRS has higher recall rates than idse-HE for the top 30 and 60 candidates. The former ranked 47.0% and 59.6% positive samples, while the latter ranked 42.1% and 58.1%, respectively. Idse-HE achieved slightly higher recall rates than GCRS for the top 90, 120, and 240 candidates. Idse-HE ranked 67.1% and 73.9% for the top 90 and 120 candidates, while GCRS ranked 66.8% and 71.9% (Figure 5). The AUC value of GCRS was very close to that of SDPred, but all of the recall rates of GCRS were higher than those of SDPred. When *k* was increased from 30 to 120, the SDPred ranked 41.8%, 54.9%, 62.3% and 67.4%, respectively. Ding’s method was not as good as SDPred, with corresponding recall rates of 35.5%, 48.2%, 56.3%, and 62.2%, respectively. The recall rates of FGRMF (32.8%, 45.2%, 52.5%, 58.1%) were slightly higher than those of Galeaon’s method (32.3%, 43.6%, 51.7%, 56.8%). The lowest recall rates were obtained by the RW-SHIN method with recall rates of 23.7%, 34.3%, 41.3% and 47.2%, respectively.

### 3.4. Case Studies on Five Drugs

According to the world mental health report in 2022, nearly one billion people across the World suffered from mental diseases. Therefore, to further demonstrate TCSD’s ability to predict drug-side-effect associations, we analyzed five psychotropic drugs, including Amitriptyline, Olanzapine, Clozapine, Aripiprazole, and Asenapine. First, using the model, we were able to obtain association scores for each drug candidate side-effect and ranked them accordingly. Then, the top 15 potential side-effects for each drug were compiled and analyzed. The results are listed in Table 3, Table 4, Table 5, Table 6 and Table 7.

MetaADEDB is a comprehensive repository of clinically reported adverse drug events (ADEs) containing 744,709 associations between 8498 drugs and 13,193 ADEs [38]. Rxlist is a searchable database of more than 5000 drugs that have appeared in physician articles and authoritative websites, such as U.S. Food and Drug Administration (FDA)-related side-effects, drug safety issues, and other bases of prescribing information [39]. Drug Central collects information on the structure, pharmacological effects, and indications of active drug ingredients approved by the FDA and other regulatory agencies, as well as on ADEs [40]. SIDER is a database of marketed drugs and their adverse reaction records, covering 5868 side-effects and 139,756 pairs of associations between 1430 drugs [28]. As shown in Table 3, 12 candidates are supported by Drug Central, 14 are included in MetaADEDB, and the Rxlist and SIDER databases also contain 14 candidates, respectively. Table 4 lists the candidates of the drug Olanzapine, and 12, 12, 15, and 15 candidates are recorded in the databases Drug Central, MetaADEDB, Rxlist, and SIDER, respectively. In addition, the constipation and vomiting of patients after they have taken the drug was confirmed by the literature [36]. We labeled these two candidates with “Literature” and added them in Table 4. As shown in Table 5 and Table 6, in terms of the drugs Clozapine and Aripiprazole, each of these two drugs has 13 candidates in Drug Central. There are 12 candidates and 15 in MetaADEDB, while Rexlist contains 12 candidates, and SIDER includes 13 candidates. In addition, dizziness and blurred vision appeared with high chance after the drug was used over 3 months [37]. The side-effect “Blurred vision” was labeled with “Literature” in Table 5. Similarly, the drug has 2, 7, 12, and 10 candidates in the four databases, respectively. Thus, TCSD has the ability to identify potential drug-related side-effect candidates. It can screen reliable candidates for biologists to undertake subsequent wet-experiment studies to determine the actual associations.

### 3.5. Predicting Novel Drug-Related Side-Effects

After we verified the predictive performance of the TCSD model, our model was utilized to predict candidate side-effects for 708 drugs, which included the drugs belonging to the antitumor, digestive, psychiatric, and nutritional categories. Biologists usually select the top-ranked candidate side-effects for biological experiments to determine the actual drug-related side-effects. We list the top 30 candidate side-effects for each of 708 drugs in the Supplementary Table S1.

## 4. Conclusions

We presented a model (TCSD), which deeply integrates the similarity and association connections with diverse semantics within multiple heterogeneous graphs for inferring potential drug-side-effect association candidates. Two constructed drug-side-effect heterogeneous graphs were beneficial for formulating their specific neighbor context encoding based on a graph-sensitive transformer. The graph-sensitive transformer also integrated the discriminative semantics from the different types of connections between a target node and its multiple kinds of neighbor nodes. A multi-layer capsule network-based module was established to capture the multi-view attribute information for each drug-side-effect node pair. Two attention mechanisms were designed to produce the more important neighbor categories and heterogeneous graph information was used to derive higher weights. The cross-validation results demonstrated TCSD’s improved prediction performance, including greater AUC and AUPR, and higher recall rates for the top-ranked candidates than the other six comparison methods. In addition, the case studies on Amitriptyline, Olanzapine, Clozapine, Aripiprazole, and Asenapine also showed TCSD’s ability in retrieving potential candidate drug-related side-effects. TCSD inferred the candidate side-effects for 708 drugs.

## Figures and Tables

**Table 1:** Performance demonstration of the ablation experiments.

CET	MVF	NCA	Average AUC	Average AUPR
✕	✓	✕	0.963	0.209
✓	✕	✓	0.971	0.254
✓	✓	✕	0.976	0.298
✓	✓	✓	**0.977**	**0.351**

**Table 2:** Results of the Wilcoxon test by comparing TCSD and the other six methods.

	GCRS	idse-HE	SDPred	Ding’s Method	FGRMF	Galeaon’s Method	RW-SHIN
*p*-value of AUC	8.4303 × 10${}^{-4}$	2.6327 × 10${}^{-4}$	4.7184 × 10${}^{-6}$	3.4493 × 10${}^{-11}$	1.8906 × 10${}^{-34}$	4.9532 × 10${}^{-41}$	2.5631 × 10${}^{-79}$
*p*-value of AUPR	2.6205 × 10${}^{-5}$	1.3362 × 10${}^{-5}$	5.3927 × 10${}^{-6}$	4.6451 × 10${}^{-14}$	2.2247 × 10${}^{-26}$	3.7876 × 10${}^{-37}$	4.8253 × 10${}^{-54}$

**Table 3:** Top 15 candidate side-effects related to Amitriptyline.

Drug	Rank	Side-Effect	Evidence	Rank	Side-Effect	Evidence
	1	Edema	Drugcentral, MetaADEDB, SIDER	9	Diarrhea	Drugcentral, MetaADEDB, Rxlist, SIDER
	2	Nausea	MetaADEDB, Rxlist, SIDER	10	Hypotension	Drugcentral, MetaADEDB, Rxlist, SIDER
	3	Vomiting	Drugcentral, MetaADEDB, Rxlist, SIDER	11	Confusion	Drugcentral, Rxlist, SIDER
Amitriptyline	4	Rash	Drugcentral, MetaADEDB, Rxlist, SIDER	12	Leukopenia	Drugcentral, MetaADEDB, Rxlist, SIDER
	5	Dizziness	Drugcentral, MetaADEDB, Rxlist, SIDER	13	Constipation	Drugcentral, MetaADEDB, Rxlist, SIDER
	6	Blurred vision	Drugcentral, MetaADEDB, Rxlist	14	Paresthesia	Drugcentral, MetaADEDB, Rxlist, SIDER
	7	Anorexia	MetaADEDB, Rxlist, SIDER	15	Syncope	MetaADEDB, Rxlist, SIDER
	8	Headache	Drugcentral, MetaADEDB, Rxlist, SIDER			

**Table 4:** Top 15 candidate side-effects related to Olanzapine.

Drug	Rank	Side-Effect	Evidence	Rank	Side-Effect	Evidence
	1	Edema	Drugcentral, MetaADEDB, Rxlist, SIDER	9	Paresthesia	Drugcentral, MetaADEDB, Rxlist, SIDER
	2	Vomiting	Rxlist, MetaADEDB, Rxlist, SIDER, Literature [36]	10	Dizziness	Drugcentral, MetaADEDB, Rxlist, SIDER
	3	Headache	Drugcentral, MetaADEDB, Rxlist, SIDER	11	Back pain	Drugcentral, MetaADEDB, Rxlist, SIDER
Olanzapine	4	Nausea	Drugcentral, MetaADEDB, Rxlist, SIDER	12	Pruritus	Drugcentral, MetaADEDB, Rxlist, SIDER
	5	Rash	Drugcentral, MetaADEDB, Rxlist, SIDER	13	Dry mouth	Rxlist, SIDER
	6	Confusion	Drugcentral, Rxlist, SIDER	14	Cough	Drugcentral, MetaADEDB, Rxlist, SIDER
	7	Diarrhea	Drugcentral, Rxlist, SIDER	15	Arthralgia	Drugcentral, MetaADEDB, Rxlist, SIDER
	8	Constipation	MetaADEDB, Rxlist, SIDER, Literature [36]			

**Table 5:** Top 15 candidate side-effects related to Clozapine.

Drug	Rank	Side-Effect	Evidence	Rank	Side-Effect	Evidence
	1	Edema	Drugcentral, MetaADEDB, Rxlist, SIDER	9	Vomiting	Drugcentral, MetaADEDB, Rxlist, SIDER
	2	Nausea	Drugcentral, MetaADEDB, Rxlist, SIDER	10	Rash	Drugcentral, MetaADEDB, Rxlist, SIDER
	3	Pruritus	Drugcentral, MetaADEDB, SIDER	11	Blurred vision	Rxlist, Literature [37]
Clozapine	4	Diarrhea	Drugcentral, MetaADEDB, Rxlist, SIDER	12	Headache	Drugcentral, MetaADEDB, Rxlist, SIDER
	5	Anemia	Drugcentral, SIDER	13	Thrombocytopenia	Drugcentral, MetaADEDB, Rxlist, SIDER
	6	Paresthesia	Drugcentral, Rxlist, SIDER	14	Nervousness	Drugcentral, MetaADEDB
	7	Pain	Drugcentral, MetaADEDB, Rxlist, SIDER	15	Dizziness	Drugcentral, MetaADEDB, Rxlist, SIDER
	8	Anorexia	MetaADEDB, Rxlist, SIDER			

**Table 6:** Top 15 candidate side-effects related to Aripiprazole.

Drug	Rank	Side-Effect	Evidence	Rank	Side-Effect	Evidence
	1	Edema	Drugcentral, MetaADEDB, Rxlist, SIDER	9	Tachycardia	Drugcentral, MetaADEDB, Rxlist, SIDER
	2	Headache	Drugcentral, MetaADEDB, Rxlist, SIDER	10	Blurred vision	Drugcentral, MetaADEDB, Rxlist
	3	Rash	Drugcentral, MetaADEDB, Rxlist, SIDER	11	Dyspepsia	Drugcentral, MetaADEDB, Rxlist, SIDER
Aripiprazole	4	Dizziness	MetaADEDB, MetaADEDB, Rxlist, SIDER	12	Chest pain	Drugcentral, MetaADEDB, Rxlist, SIDER
	5	Nervousness	Drugcentral, MetaADEDB, SIDER	13	Hemorrhage	MetaADEDB
	6	Infection	Drugcentral, MetaADEDB, Rxlist, SIDER	14	Hypersensitivity	Drugcentral, MetaADEDB, Rxlist, SIDER
	7	Constipation	Drugcentral, MetaADEDB, Rxlist, SIDER	15	Fatigue	Drugcentral, MetaADEDB, Rxlist, SIDER
	8	Back pain	Drugcentral, MetaADEDB, SIDER			

**Table 7:** Top 15 candidate side-effects related to Asenapine.

Drug	Rank	Side-Effect	Evidence	Rank	Side-Effect	Evidence
	1	Edema	MetaADEDB, Rxlist, SIDER	9	Dyspnea	Rxlist, SIDER
	2	Vomiting	Rxlist, SIDER	10	Constipation	MetaADEDB, Rxlist, SIDER
	3	Headache	MetaADEDB, Rxlist, SIDER	11	Confusion	Rxlist
Asenapine	4	Pain	MetaADEDB, Rxlist, SIDER	12	Blurred vision	unconfirmed
	5	Nausea	MetaADEDB, Rxlist, SIDER	13	Fatigue	Drugcentral, MetaADEDB, Rxlist, SIDER
	6	Dizziness	MetaADEDB, Rxlist, SIDER	14	Anorexia	unconfirmed
	7	Rash	Rxlist, SIDER	15	Pruritus	unconfirmed
	8	Diarrhea	Drugcentral, Rxlist			

## Data Availability

Not applicable.

## Supplementary Materials

The following supporting information can be downloaded at: https://www.mdpi.com/article/10.3390/molecules28186544/s1, Tablse S1: The top 30 candidate side-effects for each of 708 drugs; Table S2: The training and testing time of TCSD and the compared methods.
