# Supplementary material for: Learning Multi-Types of Neighbor Node Attributes and Semantics by Heterogeneous Graph Transformer and Multi-View Attention for Drug-Related Side-Effect Prediction
Source: Molecules. 2023 Sep 9;28(18):6544. doi: 10.3390/molecules28186544 (PMC10537290; doi:10.3390/molecules28186544)
Supplement: Supplementary file 1 [file molecules-28-06544-s001.zip › Table S2.pdf]

The training and testing time of TCSD and the compared methods was listed in the Supplementary Table ST2.

Table S2. The training and testing time of TCSD and the compared methods.

**Running Time**	**TCSD**	**GCRS**	**idse-HE**	**SDPred**	**Galeaon's Method**	**RW-SHIN**	**Ding s Method**	**FGRMF**
Average time per epoch/minute	15	17	25	5	9	20	23	11
Testing time per drug /second	0.0027	0.0030	0.0043	0.00086	0.0013	0.0050	0.00282	0.00047
